# Identification of Downregulated *MECR* Gene in Parkinson’s Disease Through Integrated Transcriptomic Analysis and Validation

**DOI:** 10.3390/ijms26020550

**Published:** 2025-01-10

**Authors:** Danlei Wang, Haoheng Yu, Yi Qu, Ke An, Hongming Liang, Zhijuan Mao, Jingyi Li, Yongjie Xiong, Zhe Min, Zheng Xue

**Affiliations:** 1 Department of Neurology, Tongji Hospital, Tongji Medical College, Huazhong University of Science and Technology, Wuhan 430030, China; 2 Department of General Practice, Tongji Hospital, Tongji Medical College, Huazhong University of Science and Technology, Wuhan 430030, China

**Keywords:** Parkinson’s disease, lipid metabolism, machine learning, drug screening, *MECR*

## Abstract

Parkinson’s disease (PD) is a neurodegenerative disorder characterized by dopaminergic neuron degeneration and α-synuclein (α-syn) aggregation. Lipid metabolism dysfunction may contribute to PD progression. This study aims to identify lipid metabolism-related genes (LMGs) associated with PD using an integrative transcriptomic analysis of microarray and single-cell RNA sequencing (scRNA-seq) datasets from patients with PD and healthy controls. Differentially expressed genes (DEGs) related to lipid metabolism were identified, and key genes were further filtered using weighted gene co-expression network analysis (WGCNA) and machine learning algorithms. Four LMGs, *AGPAT2*, *ASAH2*, *FA2H*, and *MECR* were identified, with *MECR* being notably downregulated in both bulk and single-cell transcriptomic analyses of PD patients. This downregulation was further validated in α-syn PFF-induced PD models. Virtual screening and molecular simulations identified potential allosteric modulators of *MECR*, which may offer a pathway for future therapeutic exploration. This study highlights *MECR* as a critical gene link between lipid metabolism dysfunction and PD, suggesting the need for further investigation into its therapeutic implications.

## 1. Introduction

Parkinson’s disease (PD) is a common neurodegenerative disorder marked by the gradual degeneration of dopaminergic (DA) neurons in the substantia nigra (SN), and abnormal α-synuclein (α-syn) aggregation, leading to motor dysfunction and non-motor dysfunction [1]. Although the exact etiology of PD remains unclear, genetic and environmental factors are believed to play significant roles in its pathogenesis [2].

Lipid metabolism plays a crucial role in maintaining cell membrane integrity, energy storage, and signal transduction [3,4]. Abnormalities in lipid metabolism can lead to impaired cellular function and can even induce apoptosis [5]. Dysregulation of lipid metabolism has been implicated in various neurodegenerative diseases, including PD, where it can contribute to impaired neuronal function and survival [6,7,8,9,10]. In patients with PD, changes in lipid metabolism may affect the survival and function of DA neurons, thereby accelerating disease progression [11]. For instance, abnormal aggregation of α-syn, a pathological hallmark of PD, can be influenced by lipid metabolism, affecting its conformation and aggregation [12]. Numerous genetic mutations associated with hereditary PD, including *GBA*, *LRRK2*, and *PINK1*, play significant roles in lipid metabolism [9]. However, the specific genes that are upregulated or downregulated, and the lipid metabolic pathways that are disrupted in sporadic PD, remain to be fully elucidated.

Advancements in bioinformatics have provided powerful tools for exploring the molecular mechanisms of complex diseases [13]. By analyzing single-cell sequencing data and gene expression profiles, key genes and signaling pathways related to diseases can be identified [14]. This study aims to utilize bioinformatics methods to screen key genes associated with PD and lipid metabolism, and to explore potential therapeutic drugs. This approach enhances our understanding of the molecular pathology of PD and may help identify novel molecular targets for future therapeutic interventions.

## 2. Results

### 2.1. Identification of Differentially Expressed Genes (DEGs) Between HCs and Patients with PD

**Figure 1:**
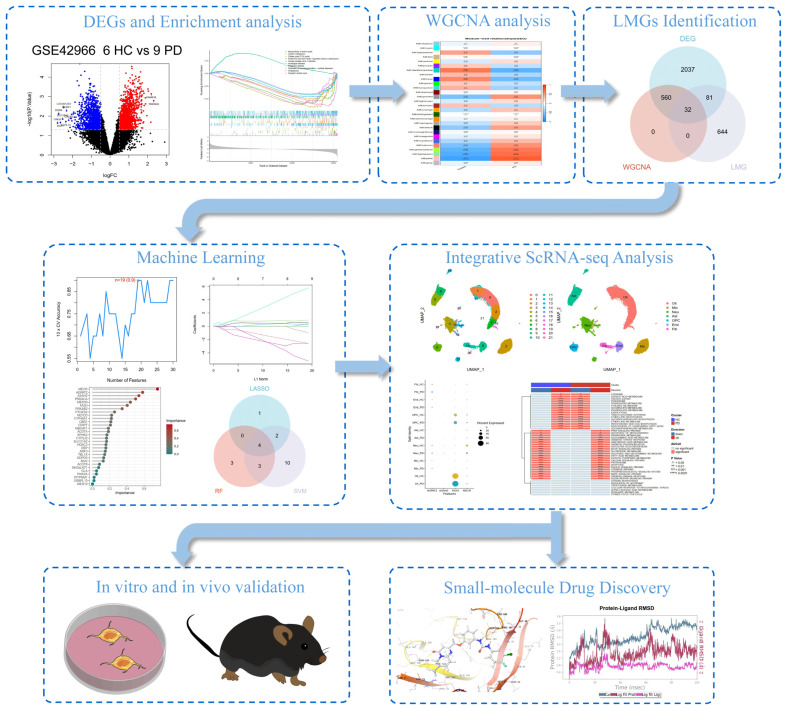
Flowchart of a multistep strategy for bioinformatics data analysis and validation.

**Figure 2:**
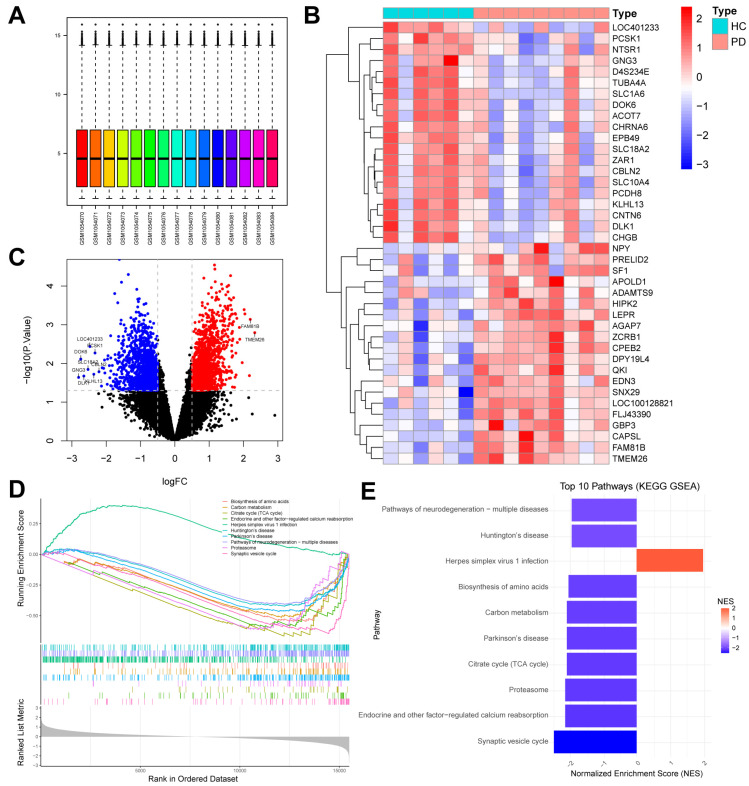
Identification of DEGs and KEGG-GSEA analysis in the GSE42966 dataset for PD. (**A**) Box plots of raw data normalized across samples from the GSE42966 dataset. (**B**) Heatmap of the top 20 most significantly upregulated (red) and downregulated (blue) genes identified in the comparison between healthy controls (HCs) and patients with Parkinson’s disease (PD). (**C**) Volcano plot showing the distribution of differentially expressed genes (DEGs). The horizontal dotted line represents a *p*-value threshold of 0.05, and the vertical dotted line represents the log FC threshold of 0.5. Blue dots represent significantly downregulated genes and red dots represent significantly upregulated genes. The top ten most significant DEGs are highlighted. (**D**,**E**) Gene set enrichment analysis (GSEA) identified the top ten pathways based on the Kyoto Encyclopedia of Genes and Genomes (KEGG) database, ranked by normalized enrich-ment score (NES).

The workflow of this study is illustrated in Figure 1. Following the normalization (Figure 2A) and differential analysis of the microarray data from the GSE42966 dataset, we identified a total of 2710 DEGs among the samples, from six healthy controls (HCs) and nine patients with PD. This included 1387 upregulated genes and 1323 downregulated genes. The heatmap depicting the top 20 most significantly upregulated and downregulated genes is presented in Figure 2B, while the volcano plot of the DEGs is shown in Figure 2C. We performed the Kyoto Encyclopedia of Genes and Genomes (KEGG) pathway enrichment analysis using the Gene set enrichment analysis (GSEA) method. In the PD group, significant downregulation was observed in pathways associated with synaptic vesicle cycle, proteasome, citrate cycle (TCA cycle), Parkinson’s disease, carbon metabolism, biosynthesis of amino acids, herpes simplex virus 1 infection, Huntington disease, and pathways of neurodegeneration-multiple diseases. Conversely, the pathway related to endocrine and other factor-regulated calcium reabsorption was significantly upregulated (Figure 2D,E). These findings highlight the neurodegenerative changes and metabolic reprogramming associated with PD.

### 2.2. Screening Key Lipid Metabolism-Related DEGs via Weighted Gene Co-Expression Network Analysis (WGCNA)

**Figure 3:**
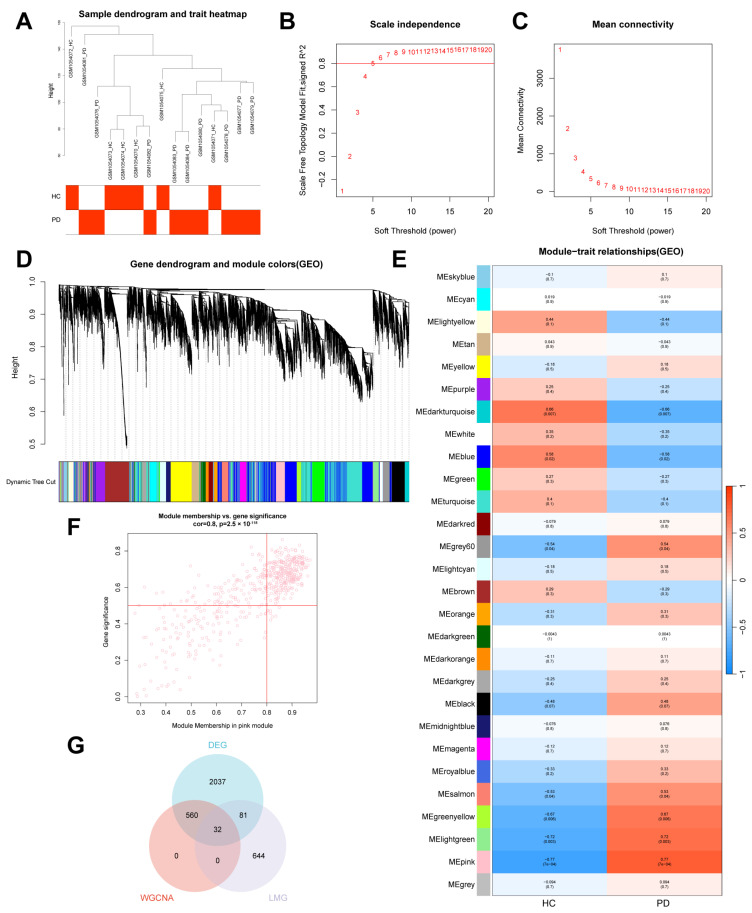
Enrichment levels in genomic weighted gene co-expression network analysis (WGCNA). (**A**) Sample clustering dendrogram with tree leaves representing each sample. (**B**,**C**) Soft threshold β = 6 and scale-free topological fit index (R2). (**D**) Initial and merged modules within the clustering tree. (**E**) Heatmap of module–trait correlations. (**F**) Scatter plot illustrating the relationship between module membership (MM) and gene significance (GS) in the pink module. (**G**) Venn diagram showing the intersection of genes identified from differentially expressed genes (DEGs), hub genes from the WGCNA, and lipid metabolism-related genes (LMGs) from the Reactome database.

We utilized WGCNA to elucidate the gene modules that are most significantly associated with disease status. All of the six HC and nine PD samples of the GSE42966 dataset were selected for clustering (Figure 3A). The optimal soft-thresholding power was determined to be six, where the scale-free topology fit index (R^2^) exceeded 0.8, and the average network connectivity was sufficiently high (Figure 3B,C). Gene modules were detected through hierarchical clustering, and the initial modules were further merged based on their eigengene similarity. The resulting clustered modules are displayed in a dendrogram (Figure 3D). Among these modules, the pink module demonstrated the strongest correlation with PD (*p* = 7 × 10^−4^) (Figure 3E). A total of 592 genes in the pink module, with module membership (MM) > 0.8 and gene significance (GS) > 0.5, were considered biologically relevant hub genes for PD (Figure 3F). Subsequently, to identify key DEGs in lipid metabolism associated with PD, 849 lipid metabolism-related genes (LMGs) from the Reactome database were intersected with the 592 hub genes identified from the pink module, through WGCNA and the DEGs, resulting in 32 overlapping genes. This overlap is illustrated in a Venn diagram (Figure 3G).

### 2.3. Identification of Key Lipid Metabolism-Related DEGs Using Machine Learning Algorithms

**Figure 4:**
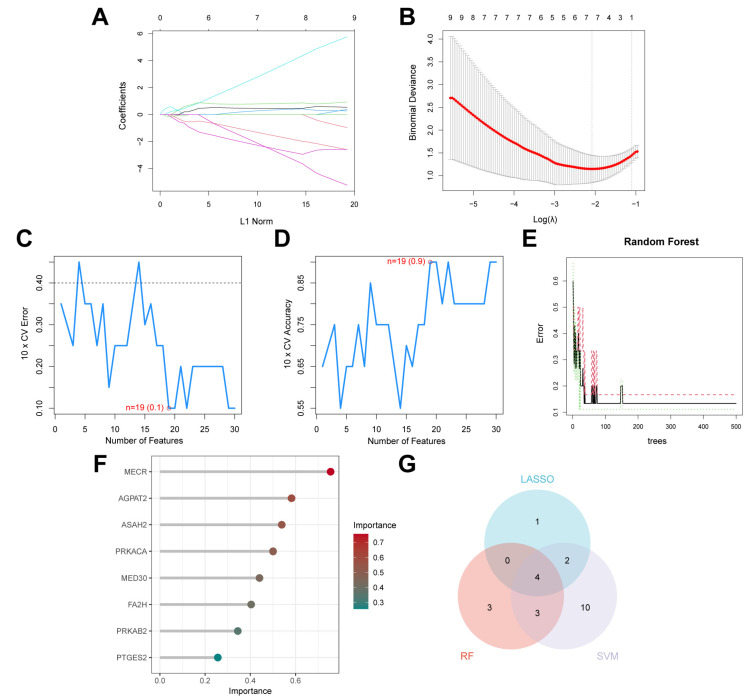
Identification of key genes using LASSO, SVM-RFE, and Random Forest algorithms. (**A**) LASSO regression plot showing the relationship between coefficients and the shrink–age parameter (λ). (**B**) Cross-validation curve for the LASSO model, used to determine the optimal λ value based on minimal deviance. (**C**) SVM-RFE cross-validation error plot displaying the error rates across different feature elimination steps. (**D**) Cross-validation accuracy plot for the SVM-RFE method, showing the accuracy at each step of recursive feature elimination. (**E**) Random Forest error plot, with the number of trees on the *x*-axis and the classification error on the *y*-axis, identifying the point where the error stabilizes. (**F**) Bubble plot showing the importance of genes identified by Random Forest, with Mean Decrease Gini > 0.25. (**G**) Venn diagram illustrating the overlap of feature genes identified by LASSO, SVM-RFE, and Random Forest algorithms.

To further identify key LMGs associated with PD, three machine learning algorithms, LASSO, SVM-RFE, and Random Forest, were applied to the previously identified 32 lipid metabolism-related DEGs. These algorithms allowed for more precise filtering of feature genes relevant to disease progression. LASSO regression identified seven genes with non-zero coefficients (Figure 4A,B). SVM-RFE, which recursively eliminates less important features, identified 19 genes with a maximum accuracy of 0.9 and a minimum error of 0.1 (Figure 4C,D). The Random Forest analysis, based on the Mean Decrease Gini index, also identified seven important genes (Figure 4E,F). The intersection of genes from all three methods yielded four candidate key genes, namely *AGPAT2*, *ASAH2*, *FA2H*, and *MECR*, which are visualized in a Venn diagram (Figure 4G). Among these, *AGPAT2*, *ASAH2*, and *MECR* were downregulated in the PD group, while *FA2H* was upregulated (Figure S1A). Moreover, we also analyzed GSE8397, a microarray dataset comprising SN samples from PD patients and controls, and observed a significant upregulation of *FA2H* expression and a pronounced downregulation of *MECR* expression (Figure S1B), consistent with our findings in the GSE42966 dataset.

To elucidate the pathways in which these four genes might be involved in PD, we used the GSEA method to analyze the correlation between the expression of these genes and KEGG pathways (Figure S1C–F). The results showed that pathways such as proteasome, synaptic vesicle cycle, oxidative phosphorylation, PD, Huntington disease, prion disease, and spinocerebellar ataxia were significantly positively correlated with *AGPAT2*, *ASAH2*, and *MECR*, and significantly negatively correlated with *FA2H*, suggesting that these four genes may play essential roles in protein degradation, neural signal transmission, energy metabolism, and neurodegenerative diseases.

### 2.4. Downregulation of MECR in Patients with PD at the Single-Cell Level

**Figure 5:**
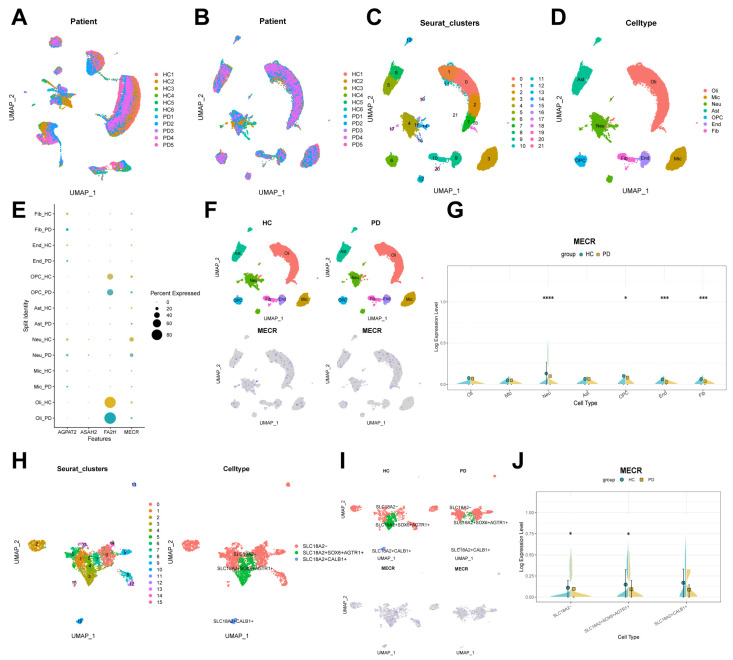
ScRNA-seq UMAP clustering, cell annotation, and key genes expression in PD. (**A**,**B**) Uniform Manifold Approximation and Projection (UMAP) plot showing cell distribution before (**A**) and after (**B**) batch effect correction using Harmony. (**C**) UMAP-based clustering identifies 22 distinct cell clusters. (**D**) UMAP plot depicting the annotation of seven cell types based on marker genes. (**E**) Dot plot showing the expression levels of candidate key genes across different cell types. (**F**) Feature plots showing *MECR* expression across various cell types, comparing healthy controls (HCs) and Parkinson’s disease (PD) groups. (**G**) Violin plot demonstrating the differential expression of *MECR* between PD and HC groups across different cell types. (**H**) UMAP plots displaying 16 distinct cell clusters in neurons (**left**) and the annotation of three neuron subtypes based on marker genes (**right**). (**I**) Feature plots highlighting *MECR* expression in neurons, comparing HC and PD groups. (**J**) Violin plot demonstrating the differential expression of *MECR* between PD and HC groups across different neuron subtypes. Significance was tested using Wilcoxon Rank Sum test. * *p* < 0.05, *** *p* < 0.001, **** *p* < 0.001.

To validate the expression differences of candidate key genes between PD and HC groups at the single-cell level, we analyzed the single-cell RNA sequencing (scRNA-seq) dataset GSE157783, which includes six HC samples and five PD samples. Initially, Uniform Manifold Approximation and Projection (UMAP) dimensionality reduction, without batch effect correction, revealed a clear separation of cells from different batches (Figure 5A). Post-correction, using Harmony package, the distribution of cells in UMAP space became more uniform, effectively eliminating inter-batch differences (Figure 5B). Subsequently, clustering analysis identified 22 distinct cell clusters (Figure 5C). Based on known marker genes (Figure S2A), these clusters were annotated into seven major cell types: oligodendrocytes, microglia, neurons, astrocytes, oligodendrocyte precursor cells (OPCs), endothelial cells, and fibroblasts (Figure 5D). The dot plots of the overall expression of the four key genes (*AGPAT2*, *ASAH2*, *FA2H*, and *MECR*) between the HC and PD groups is shown in Figure S2B–E. Notably, MECR expression significantly decreased in PD patients, consistent with microarray results (Figure S2E). *MECR* encodes mitochondrial trans-2-enoyl-CoA reductase involved in mitochondrial fatty acid synthesis [15,16]. Dot plots and feature plots of those candidate genes showcased differences in their expression patterns across various cell types and between HC and PD groups (Figure 5E,F and Figure S2F). Specifically, we observed that *MECR* was downregulated across multiple cell types in patients with PD (Figure 5G,H). In particular, *MECR* expression was significantly reduced in neurons (*p* = 1.06 × 10^−7^), OPCs (*p* = 0.029), endothelial cells (*p* = 2.9 × 10^−4^), and fibroblasts (*p* = 0.009) in patients with PD (Figure 5G). These findings suggest that *MECR* expression may be altered in PD and that its downregulation could potentially reflect changes in multiple cell types.

To explore the expression of candidate key genes in DA neurons, we extracted neurons from the GSE157783 dataset and identified 16 clusters (Figure 5H, left). Given that ventral SN DA neurons are more susceptible to PD-related neurodegeneration compared to dorsal DA neurons [17], we used *SLC18A2* and *CALB1* to label dorsal DA neurons, and *SLC18A2*, *SOX6*, and *AGTR1* to label ventral DA neurons, thereby dividing the neurons into three distinct cell subtypes: *CALB1*+ DA neurons, *SOX6*+*AGTR1*+ DA neurons, and non-DA neurons (Figure S2G; Figure 5H, right). The number of *SOX6*+*AGTR1*+ DA neurons was reduced in PD patients (Figure 5I), and *MECR* expression significantly decreased in *SOX6*+*AGTR1*+ DA neurons from PD patients (Figure 5I,J; *p* = 0.022). However, no significant differences were observed in the expression of *AGPAT2*, *ASAH2*, and *FA2H* in this subtype. This suggests that the decreased expression of MECR may be closely associated with neurodegeneration in PD.

### 2.5. Single-Cell Insights into Lipid/Energy Metabolism Reprogramming and Oxidative Damage in PD

Given that previous studies have suggested *MECR* deficiency leads to impaired mitochondrial fatty acid synthesis, affecting mitochondrial function and resulting in energy metabolic dysfunction and oxidative stress [18], we conducted single-cell GSEAs to investigate alterations in lipid metabolism, energy metabolism, and oxidative stress pathways in the scRNA-seq of PD patients. Overall, lipid synthesis and metabolic pathways exhibit a downregulation trend in PD compared to HCs, both at the overall level and across multiple cell types. This includes the synthesis of unsaturated fatty acids, ether lipids, glycerophospholipids, phospholipids, and triglycerides (Figure S3A). These pathways are significantly downregulated in one or more major cell types, while steroid biosynthesis does not show significant differences (Figure S3A–F). Additionally, the metabolic processes of linoleic acid, glycerolipid, glycerophospholipid, sphingolipid, and phospholipid are overall downregulated in PD. Although there is no overall difference in α-linolenic acid and arachidonic acid, they are downregulated in several cell types. However, fatty acid metabolism is upregulated in both the overall sample and microglia (Figure S3A–F). Furthermore, energy metabolism is found to be downregulated in several major cell types, including the synthesis of Coenzyme A (CoA) and pantothenate, pyruvate metabolism, and the TCA cycle (Figure S3A–F). In contrast, apoptosis, oxidative stress, oxidative damage, calcium signaling, and phosphoinositide metabolism pathways are upregulated both overall and across various cell types in PD patients (Figure S3A–F). We also analyzed *SOX6*+*AGTR1*+ DA neurons, and the results showed a decrease in glycerophospholipid and phospholipid metabolism, while oxidative damage response, calcium signaling, and phosphoinositide metabolism pathways were upregulated, consistent with the overall trend (Figure S3A–G).

### 2.6. Validation of MECR Downregulation in α-Syn PFF-Induced Models

**Figure 6:**
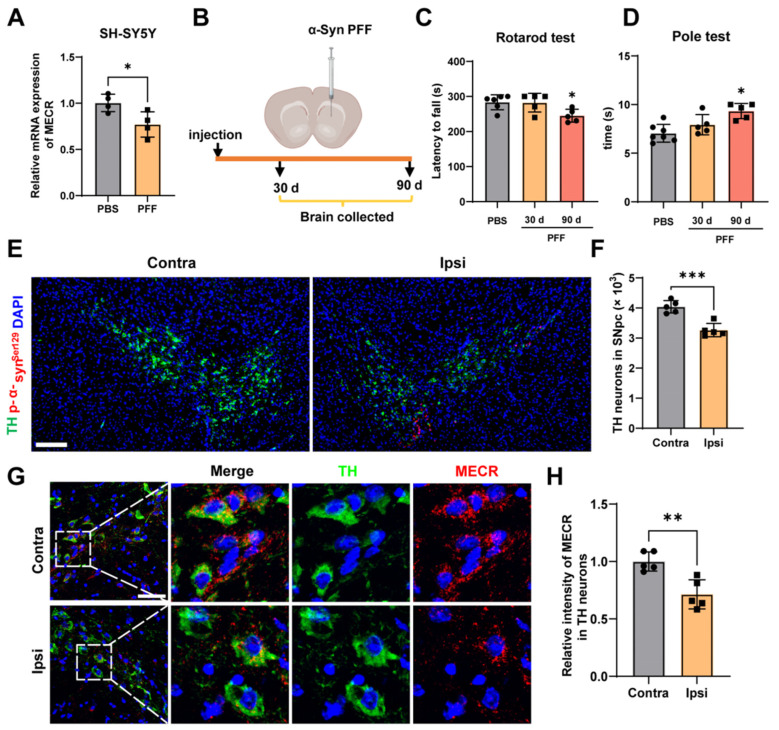
MECR is downregulated in PD neurons induced by α-syn PFFs. (**A**) qPCR analysis showing *MECR* mRNA expression in SH-SY5Y cells treated with PBS or α-syn PFFs. N = 4 per group. (**B**) Schematic of the α-syn PFF-injected mouse model. (**C**) Rotarod test results showing the latency to fall at 30- and 90-days post-α-syn PFF injection. N = 5–6 per group. (**D**) Pole test results showing the descending time at 30- and 90-days post-α-syn PFF injection. N = 5–7 per group. (**E**) Representative confocal images of TH (green), p-α-synSer129 (red) and DAPI (blue) staining in the contralateral (Contra) and ipsilateral (Ipsi) sides of the SN 90 days post-injection. Scale bar: 200 μm. (**F**) Quantification of TH+ cells in the SN. N = 4 per group. (**G**) Confocal images of TH (green), MECR (red) and DAPI (blue) staining in the contralateral and ipsilateral sides of the SN 90 days post-injection. Scale bar: 20 μm. (**H**) Quantification of MECR protein intensity in TH+ cells in the SN. Data are presented as means ± SEMs. Significance in (**A**,**F**,**H**) was tested with a two-tailed unpaired Student’s *t*-test. Significance in (**C**,**D**) was tested using one-way ANOVA. * *p* < 0.05, ** *p* < 0.01, *** *p* < 0.001.

To validate the downregulation of MECR in PD DA neurons, we employed both cellular and mouse models induced by α-syn PFFs. Quantitative PCR (qPCR) analysis in SH-SY5Y cells confirmed a significant reduction in *MECR* mRNA expression in the α-syn PFF-treated group compared to the PBS control (Figure 6A). In the mouse model, motor deficits were observed only at 90 days post-injection, with a significant decrease in the latency to fall in the Rotarod test and an increased descending time in the Pole test (Figure 6C,D). Therefore, subsequent fluorescence detection was conducted on 90-day PFF mice. Immunofluorescence staining in the SN of 90-day PFF mice showed a marked reduction in TH+ neurons on the ipsilateral side, alongside the spread of p-α-syn^Ser129^, compared to the contralateral side (Figure 6E,F). MECR protein levels were also significantly lower in TH+ neurons on the ipsilateral side (Figure 6G,H). These results confirm that MECR is downregulated in both the α-syn PFF-induced cellular and 90-day mouse models, correlating with motor impairments, TH+ neuronal loss, and p-α-syn^Ser129^ spread in the SN.

### 2.7. Prediction of Potential Allosteric Modulators of MECR

**Figure 7:**
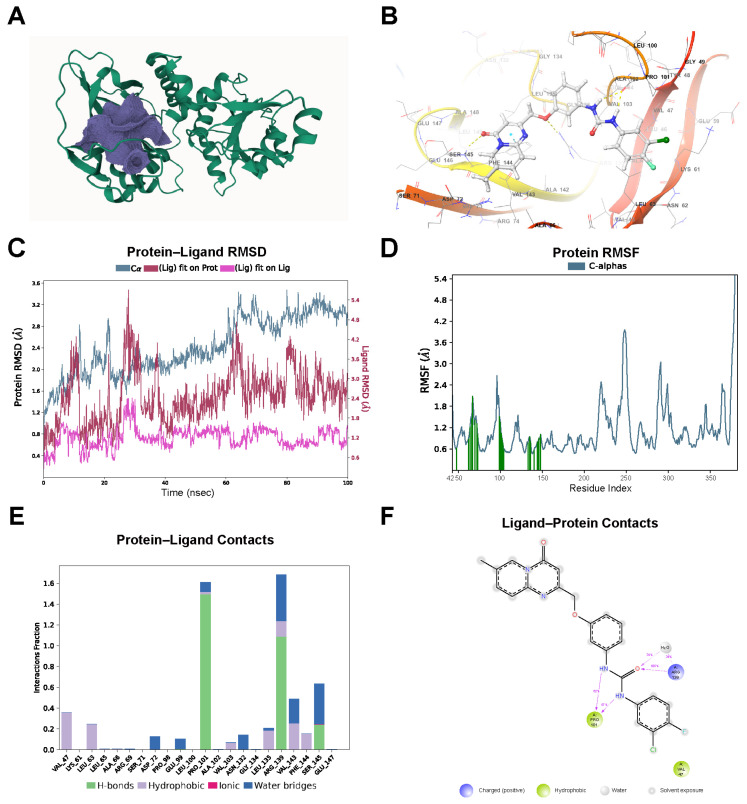
Identification and stability assessment of allosteric modulators for MECR. (**A**) A 3D structural simulation of the MECR protein (green) and the predicted allosteric pocket (purple). (**B**) Molecular docking diagram of the candidate compound M-03 binding with MECR. (**C**) Root mean square deviation (RMSD) analysis of the MECR–M-03 complex from 100 ns molecular dynamics (MDs) simulations. (**D**) Protein Root Mean Square Fluctuation (RMSF) plot of MECR, with protein residues interacting with the compound M-03 indicated by green vertical bars in the diagram. (**E**) Protein–ligand contacts bar plot of the MECR protein and the compound M-03. (**F**) Interaction diagram between the compound M-03 and the protein MECR.

Since *MECR* mutations can lead to mitochondrial dysfunction and neurodegeneration [19,20,21], and our transcriptomic analysis (microarray and scRNA-seq) has shown decreased *MECR* expression in PD, we aimed to screen for potential allosteric activators of MECR as candidates for further investigation in PD. Structural analysis of the MECR protein (PDB: 7AYB) using the CavityPlus platform revealed multiple potential binding pockets. Following further evaluation, the pocket comprising VAL-47, GLU-107, ASN-88, MET-89, LEU-135, VAL-103, GLY-92, ALA-45, GLY-134, THR-137, HIS-51, GLY-105, PHE-144, GLY-136, LEU-100, GLY-104, ILE-90, ALA-133, MET-288, ALA-148, GLY-95, LEU-97, ASN-106, GLN-91, ASN-132, GLY-287, TYR-48, ASP-86, SER-85, ARG-139, ASN-93, LYS-316, ALA-102, ALA-131, PRO-101, PRO-98, LEU-96, GLU-99, TYR-94, LEU-63, and LEU-46 (Figure 7A) was assessed with strong druggability and a DrugScore of 2644.00. This pocket exhibits favorable binding characteristics, making it suitable as an allosteric site for subsequent drug screening.

Based on the selected allosteric pocket, virtual screening was conducted using the Glide module in Schrödinger software (Schrödinger Release 2023–2) on the ChemDiv database. Compounds were filtered through three steps: HTVS, SP, and XP screening, with additional criteria including State Penalty = 0, MMGBSA ΔG Bind < −50 kcal/mol, and Ligand Strain Energy < 10 kcal/mol, resulting in six candidate compounds, designated as M-01 to M-06 (Table 1, Figure S4A). Following these numerical threshold filters, further manual selection was conducted by analyzing hydrogen bonding, hydrophobic interactions, and other non-covalent interactions between the ligands and protein. Three compounds, M-01, M-02, and M-03 met the selection criteria, demonstrating stable hydrogen bonding, hydrophobic interactions, and good spatial and chemical compatibility within the pocket (Table 1).

Subsequent 100 ns molecular dynamics (MD) simulations of the candidate compound–protein complexes were performed using Desmond. Analysis of the compound M-03–protein complex (Figure 7B) indicated that the protein’s RMSD stabilized after 80 ns (Figure 7C). Although the ligand initially exhibited relatively high RMSD fluctuations, the amplitude gradually decreased after 80 ns, indicating improved stability despite ongoing fluctuations. Video trajectory (Video S1) demonstrated a more stable conformation and binding mode for compound M-03 compared to the other two compounds (Figure S4B,C; Videos S2 and S3). The per-residue root mean square fluctuation (RMSF) values for the protein are shown in Figure 7D. The maximum fluctuation at ligand-binding residues was 2.2 Å, with most binding site residues under 1.5 Å, indicating stability at the ligand binding site. Figure 7E,F shows significant hydrogen bonding, hydrophobic interactions, and water bridges between the protein and ligand across multiple residues, with the most frequent hydrogen bond (149%) observed at Pro-101, followed by Arg-139, which also formed notable hydrogen bonds (106%) and the most water bridges (68%), thus ensuring the stability of the protein–ligand complex.

## 3. Discussion

This study combines transcriptomics and machine learning analysis to identify *MECR* as a key lipid metabolism gene potentially involved in sporadic PD. The decreased expression level of *MECR* in patients with PD was further validated through scRNA-seq data. Additionally, this finding was confirmed in the neuron cell line and mouse model treated with α-syn PFFs. Using computational simulation, we predicted potential allosteric sites for MECR and screened for allosteric modulators that could stably bind to MECR. These findings suggest that MECR, and its potential modulators, warrant further investigation as exploratory targets for understanding PD pathogenesis and developing future therapeutic strategies.

We analyzed microarray datasets from the SN of PD and HC samples, with GSEA results indicating that pathways related to synaptic vesicle cycling, the proteasome, and energy metabolism are downregulated in PD. Since lipids are essential components of cellular organelles [5], and play critical roles in neurotransmission [22,23], protein degradation [24], and energy metabolism [25], we hypothesize that disruptions in lipid metabolism may be a key factor contributing to PD pathology. Therefore, in this study, we intersected the 849 genes from the lipid metabolism gene set in the Reactome database with the DEGs, and combined WGCNA and machine learning analysis to identify four key lipid metabolism genes associated with PD: *AGPAT2*, *ASAH2*, *FA2H*, and *MECR*. GSEA also revealed that the expression of these genes is also significantly correlated with pathways involved in neurotransmission, protein degradation, and energy metabolism. *AGPAT2* encodes 1-acylglycerol-3-phosphate O-acyltransferase 2, an enzyme involved in glycerophospholipid biosynthesis [26]. The downregulation of *AGPAT2* may contribute to the observed reduction in glycerophospholipid levels in PD, affecting membrane composition and cellular signaling. *ASAH2*, which encodes neutral ceramidase, is involved in the metabolism of ceramides [27], a class of lipids that play a role in cell signaling, apoptosis, and inflammation [28,29,30]. Dysregulation of ceramide metabolism has been associated with neurodegenerative diseases [18,31]. Consequently, the downregulation of *ASAH2* in PD may result in ceramides accumulation, thereby promoting inflammation and neuronal apoptosis. *FA2H* encodes fatty acid 2-hydroxylase, an enzyme involved in the 2-hydroxylation of the N-acyl chain of ceramide to form various sphingolipids [32]. *FA2H* ablation increases intracellular ceramide and glucoceramide abundance [33], and it can lead to neurodegeneration with brain iron accumulation [34]. In this study, *FA2H* expression is upregulated in PD, which may be due to a compensatory response triggered by ceramide accumulation in PD. The dysregulation of lipid metabolism involving *AGPAT2*, *ASAH2*, and *FA2H* may collectively contribute to the pathogenesis of PD. Unfortunately, we did not observe the same expression patterns for *AGPAT2*, *ASAH2*, and *FA2H* in the GSE157783 scRNA-seq results as we observed in the GSE42966 datasets. The differences in expression patterns of *AGPAT2*, *ASAH2*, and *FA2H* between the two datasets are likely due to variations in experimental platforms and sample types. GSE42966 uses bulk tissue from the SN, which represents an average expression profile across all cell types, while GSE157783 is based on scRNA-seq, which captures the heterogeneity of gene expression in different cell populations. Additionally, patient-specific factors, such as disease stage or treatment, may also contribute to these discrepancies. Therefore, the expression and roles of these genes in PD require further investigation.

Among the four key genes identified, *MECR* emerged as a particularly important gene, showing significant downregulation in both bulk and single-cell transcriptomic analyses. *MECR* encodes mitochondrial trans-2-enoyl-CoA reductase, a key enzyme in the mitochondrial fatty acid synthesis (mtFAS) pathway, involved in the final reaction step of mitochondrial fatty acid production [15,16,35]. mtFAS is an essential pathway for the production of lipoic acid [35], which serves as a cofactor for metabolic enzymes in the mitochondrial TCA cycle [36]. Loss of MECR function results in the deficiency of mitochondrial lipoylated proteins, leading to an impaired TCA cycle, reduced activity of respiratory complex I, increased reactive oxygen species (ROS) production, and promotion of cellular oxidative damage [37]. MECR-deficient cells may lead to HIF-1α degradation and ROS accumulation under hypoxic conditions, exacerbating cellular damage [38]. In neurons, MECR deficiency causes dysregulation of mitochondrial iron metabolism, leading to an increase in ROS and ceramides, which results in neurodegeneration [18]. Mutations in human *MECR* cause severe neurodegeneration, often manifesting as childhood-onset dystonia, basal ganglia degeneration, and optic atrophy, collectively referred to as mitochondrial enoyl-CoA reductase protein-associated neurodegeneration (MEPAN) [19,20,21]. Given the crucial role of MECR in maintaining mitochondrial function, its downregulation may have widespread consequences, especially in cells with high energy demands, such as neurons. Mitochondrial dysfunction is a well-established hallmark of PD [10]. Neurons in high-energy-demanding states, particularly DA neurons, are especially vulnerable to damage, experiencing mitochondrial dysregulation that leads to reduced ATP production and oxidative stress-related damage [10]. In scRNA-seq analysis, *MECR* shows the most significant decrease in neurons from PD patients, and is also significantly downregulated in the vulnerable subpopulation of ventral DA neurons (*SLC18A2*+*SOX6*+*AGTR1*+), suggesting that *MECR* may contribute to impaired mitochondrial function, resulting in reduced energy production and heightened neuronal vulnerability to stress.

Beyond neurons, *MECR* is also downregulated in multiple cell types, including OPCs, endothelial cells, and fibroblasts. In OPCs, *MECR* downregulation could impair their ability to mature into myelinating cells, leading to compromised myelin production and subsequent axonal dysfunction [39]. Endothelial cells and fibroblasts, responsible for maintaining the blood–brain barrier [40], may also be affected, potentially disrupting vascular homeostasis and contributing to neuroinflammation. This widespread downregulation implies that *MECR* may play a broader role in maintaining cellular function beyond neurons, potentially impacting the entire cellular microenvironment within the SN.

Since we hypothesize that *MECR* might lead to the downregulation of mitochondrial energy metabolism, as well as oxidative stress, we performed single-cell GSEA on the scRNA-seq data. Our findings revealed an overall downregulation of energy metabolism, including the TCA cycle, pyruvate metabolism, and the synthesis of CoA and pantothenic acid. Concurrently, pathways related to ceramide, apoptosis, calcium signaling, oxidative stress, and oxidative damage were upregulated. This aligns with our expectations as MECR dysfunction disrupts the TCA cycle, leading to reduced energy metabolism and accumulation of ROS, ultimately causing oxidative damage and cell apoptosis. Additionally, we observed an overall trend of reduced lipid metabolism in PD, including unsaturated fatty acids, ether lipids, glycerophospholipids, and triglycerides. Previous studies have also shown that these alterations in lipid metabolism are associated with PD [41,42,43]. Given the minimal contribution of the mtFAS pathway to cellular lipid synthesis [35], we propose that this reprogramming of lipid metabolism may be independent of *MECR* dysfunction and may jointly contribute to the pathogenesis of PD.

Currently, no studies have investigated MECR expression in sporadic PD. Given that *MECR* is predominantly downregulated in neurons, we further validated its expression in neuronal models of PD, including animal and cellular models. In the SH-SY5Y cell model, treatment with α-syn PFFs led to a marked reduction in MECR expression. Similarly, in the α-syn PFF mouse model, MECR expression was significantly decreased in DA neurons of the substantia nigra, which correlated with motor deficits and the spread of phosphorylated α-syn. These findings suggest that MECR downregulation may be a critical event linking lipid metabolism dysregulation to neuronal loss and motor symptoms in PD.

Given that MECR downregulation has been observed in PD patients as well as in animal and cellular models, and that MECR deficiency can lead to neurodegeneration [19,20,21], we propose that MECR may be a potential target for further investigation in mitigating neurodegeneration associated with PD. Although some studies have suggested that lipoic acid can alleviate the effects of MECR deficiency [44], others indicate that exogenous lipoates are ineffective at rescuing lipoate deficiency and neurodegeneration [35,37,45]. Therefore, we aimed to explore compounds capable of modulating MECR activity through allosteric activation. Using the CavityPlus platform, we identified druggable allosteric pockets within the MECR protein structure. Virtual screening through Schrödinger’s Glide module resulted in six candidate compounds, with three (M-01, M-02, and M-03) showing promising interactions. Molecular dynamics simulations further indicated compound M-03 as the most stable candidate, demonstrating significant protein–ligand interactions and binding site stability. These findings suggest that compound M-03 holds promise as a potential modulator of MECR activity and warrants further experimental validation to evaluate its ability to restore mitochondrial function and mitigate PD-related cellular dysfunction. Future studies will focus on experimentally validating these compounds to better understand their impact on MECR function and their potential therapeutic implications for PD.

This study combined transcriptomics, machine learning, and computational simulations to identify MECR as a potential target for PD. MECR had not been previously studied in the context of sporadic PD, and this research is the first to propose a link between MECR downregulation and PD, with validation in both cell lines and animal models. The identification of allosteric sites on MECR, and subsequent virtual screening of candidate activators, have highlighted compounds with potential modulatory effects on MECR activity, suggesting their promise for further investigation as the therapeutic candidates aimed at restoring mitochondrial function in PD. However, some limitations must be acknowledged. First, while the integration of microarray and scRNA-seq data provided complementary insights, technical variability and batch effects may still introduce biases. Second, the identified candidate compounds remain computational predictions, and their efficacy and safety require in vitro and in vivo validation in PD models. Third, although MECR downregulation was validated across datasets and experimental models, the causal relationship between MECR deficiency and PD pathology remains unclear, necessitating functional studies such as gene knockout and overexpression experiments. In conclusion, while this study provides valuable insights into the role of MECR in PD and identifies promising candidate compounds for MECR activation, further experimental and translational research is essential to fully validate these findings and explore their therapeutic potential.

## 4. Materials and Methods

### 4.1. Datasets Acquisition

The transcriptomic datasets GSE42966 and GSE157783 were obtained from the NCBI GEO database (http://www.ncbi.nlm.nih.gov/geo, accessed on 15 August 2024). Microarray dataset GSE42966 was generated using the GPL4133 platform and includes SN samples from 6 HCs and 9 patients with PD. Microarray dataset GSE8397 was generated using the GPL96 platform and includes 15 HC SN samples and 24 PD SN samples. ScRNA-seq dataset GSE157783 was generated using the GPL24676 platform and consists of SN samples from 6 HCs and 5 patients with PD.

### 4.2. DEGs Identification and Enrichment Analysis of Microarray Data

The microarray gene expression data were normalized and analyzed for differential expression using the “limma” package in R (version 4.4.1). DEGs were identified with a log fold-change threshold of 0.5 and an *p*-value threshold of 0.05. Data visualization was carried out using the “pheatmap” package version 1.0.12 for heatmaps and “ggplot2” for volcano plots.

GSEA was performed using the “clusterProfiler” package version 4.12.6, with pathway enrichment assessed using the KEGG database. A significance threshold of *p*-value < 0.1 was applied. The top ten significantly changed pathways were selected based on their normalized enrichment scores (NES), and GSEA plots were generated for these pathways using the gseaplot2 function.

### 4.3. WGCNA

The WGCNA analysis was conducted using the “WGCNA” and “impute” packages in R. Initially, a sample clustering analysis was performed to detect and exclude outliers, enhancing the robustness of the network analysis. Next, an optimal soft-thresholding power was selected to construct a network with desirable scale-free topology. This power was chosen based on achieving an R^2^ close to 0.8 and a mean connectivity near zero, thus prioritizing network specificity and biological relevance. Gene similarity and adjacency were then computed to build a weighted network structure. Gene modules were identified using hierarchical clustering combined with a dynamic tree-cutting algorithm, with each module color-coded for easy visualization. Correlation analysis was performed to examine the relationship between modules and external traits, calculating module–trait correlations and their significance. For each gene, module membership (MM) and gene significance (GS) were computed, quantifying the gene’s representativeness within its module and its correlation with clinical traits, respectively. Genes in the module with the strongest module–trait correlation that showed significant associations with clinical traits (MM > 0.8 and GS > 0.5) were considered biologically relevant. Among these, hub genes were identified based on high intramodular connectivity, underscoring their central role within the module and potential relevance to disease mechanisms. This approach facilitated the selection of key gene modules and hub genes for further in-depth analysis and validation.

### 4.4. Identification of Feature Genes Using Machine Learning Algorithms

Three machine learning algorithms, Least Absolute Shrinkage and Selection Operator (LASSO) regression [46], Support Vector Machine Recursive Feature Elimination (SVM-RFE) [47], and Random Forest [48], were employed to identify feature genes. The input data consisted of 32 differentially expressed lipid metabolism-related genes (DEGs) identified from transcriptomic datasets. Genes were classified based on their expression patterns in PD and HC samples. The LASSO model was constructed, applying cross-validation to determine the optimal regularization parameter (λ). Non-zero coefficients from the LASSO model were selected as potential feature genes. The SVM-RFE method was then utilized to further refine the selection by constructing an SVM model and recursively eliminating less important features, ultimately selecting the top k important genes based on their importance scores. Additionally, the Random Forest algorithm was implemented to assess gene importance, building a Random Forest model and calculating each gene’s contribution to the classification results. Genes were ranked based on the Mean Decrease Gini index, with those having importance scores greater than 0.25 selected as feature genes. Each algorithm was validated using internal cross-validation to ensure robust performance and reduce overfitting. Finally, an intersection analysis of the genes identified by all three algorithms was performed to obtain the final key genes believed to play significant roles in disease progression.

### 4.5. ScRNA-Seq Data Processing and Analysis

We obtained the scRNA-seq dataset GSE157783 from the GEO database and conducted the analysis using R. Quality control was applied, during which mitochondrial and ribosomal genes were excluded, and cells with nFeature_RNA between 500 and 7500 were retained. Data normalization was performed with the Seurat package, and 2000 highly variable genes (HVGs) were identified. Dimensionality reduction was carried out using principal component analysis (PCA), with the first 30 principal components (PCs) selected. To correct for batch effects, the Harmony package was utilized with dims = 30. The batch-corrected data were then visualized and clustered using UMAP, with cell types being annotated according to established neuronal marker genes. Following cell type annotation, differential expression analysis was performed between the HC and PD groups within each cell type, identifying key lipid metabolism-related DEGs across multiple cell types, providing valuable insights into gene expression dynamics in PD. Additionally, single-cell GSEA of scRNA-seq data was conducted using the AUCell method from the “irGSEA” R package version 3.2.6 [49], and heatmaps were generated to visualize the results.

### 4.6. SH-SY5Y Cells Culture and Treatment

SH-SY5Y cells were cultured in DMEM/F12 medium (Boster Bio, Wuhan, China) supplemented with 10% fetal bovine serum (FBS, Boster Bio) and maintained in a 37 °C incubator with carbon dioxide. The cells were passaged every three days. For the intervention, human α-syn pre-formed fibrils (α-syn PFFs) were prepared from human α-syn monomers (Novoprotein, Suzhou, China) at a concentration of 5 mg/mL, incubated in a constant-temperature shaker at 37 °C for 7 days. SH-SY5Y cells were then treated with α-syn PFFs at a final concentration of 1 mg/mL for 24 h, ensuring that the α-syn PFFs were sonicated before application.

### 4.7. qPCR Analysis

Total RNA was extracted from SH-SY5Y cells using the RNAex Pro RNA extraction reagent (Agbio, Changsha, China). RNA reverse transcription and qPCR were conducted utilizing the 1st Strand cDNA Synthesis SuperMix kit and the qPCR SYBR Green Master Mix kit (Yeasen Biotechnology, Shanghai, China). The primers used for mRNA amplification (Tsingke Biotechnology, Beijing, China) were as follows: MECR (Forward: AGAAGATATGTGGGGTGCTGT; Reverse: TTTGTGAGAGCCCACCTTCA) and β-actin (Forward: CATGTACGTTGCTATCCAGGC; Reverse: CTCCTTAATGTCACGCACGAT). qPCR amplifications were carried out on a CFX Connect Detection System (Bio-Rad, Hercules, CA, USA) under the following conditions: initial denaturation at 95 °C for 3 min, followed by 40 cycles of 10 s at 95 °C, and 30 s at 55 °C. The relative gene expression levels were calculated using the 2^−ΔΔCt^ method, with β-actin as the internal control.

### 4.8. Establishment of α-Syn PFF Mouse Model

C57BL/6J male mice (specific pathogen-free, 10 weeks old) were obtained from Gempharmatech (Nanjing, China). The mice were housed under standard laboratory conditions, which included a 12-h light/dark cycle, controlled temperature (23 ± 2 °C), and unrestricted access to food and water. All animal studies received approval from the Animal Ethics Committee at Tongji Hospital. Prior to the experiments, the mice were allowed to acclimate to the laboratory environment for at least one week.

For the establishment of the α-syn PFF mouse model, the preparation of mouse α-syn PFFs followed the same protocol as human α-syn PFFs, as previously described, and was subjected to ultrasonic disruption before use. Mice were anesthetized with isoflurane and secured in a stereotactic frame. A total of 5 μL of α-syn PFF solution (1 mg/mL) was injected into two sites of the right striatum at a rate of 0.4 μL/min using a microsyringe (Hamilton, Reno, NV, USA). The coordinates for the injection sites were AP +0.6 mm, ML −2.0 mm, DV −3.7 mm, and AP 0 mm, ML −2.3 mm, DV −3.5 mm, relative to bregma. After the injection, the needle was left in place for 5 min to ensure diffusion and prevent reflux along the injection track before being slowly withdrawn.

### 4.9. Behavioral Tests

To evaluate motor coordination and bradykinesia, mice were subjected to both the Rotarod test and the Pole test. For the Rotarod test, mice were placed on a rotating rod (SANS Biotechnology, Nanjing, China) and trained at a constant speed of 10 rpm for 10 min, three times a day, over a period of three days prior to the formal experiment. In the formal test, the rod accelerated from 4 rpm to 60 rpm over a period of 300 s. The time each mouse remained on the rod before falling was recorded as the latency to fall. The average latency from three trials was calculated for each mouse.

For the Pole test, mice were positioned head-up at the top of a vertical pole (50 cm high, 1 cm diameter, rough-surfaced to provide grip). Mice were trained for three consecutive days before the formal experiment, descending from the top to the base of the pole three times per day to ensure they could complete the task. In the formal test, the time it took for each mouse to turn completely head-down and descend to the base was measured. Each mouse underwent five trials, and the average descent time was calculated.

### 4.10. Frozen Sectioning and Immunofluorescence (IF) Staining

Mice were euthanized using isoflurane and then underwent transcardial perfusion with 30 mL of precooled PBS, followed by 20 mL of precooled 4% paraformaldehyde (PFA). The brain was carefully extracted, ensuring minimal structural damage. The collected brain tissue was then fixed in 4% PFA overnight, followed by dehydration in 30% sucrose solution for 3 days.

The tissue was embedded in OCT compound and rapidly frozen in liquid isopentane for cryosectioning. Frozen tissue was sectioned into thin coronal slices (14 μm) using a freezing microtome (Thermo Fisher Scientific, Waltham, MA, USA).

For IF, sections were first immersed in citrate-based antigen retrieval solution (Beyotime, Shanghai, China) at 95 °C for 25 min. After cooling to room temperature and washing with PBS, blocking and permeabilization were performed using a blocking solution containing Triton X-100 (Beyotime) for 15 min. The sections were subsequently incubated overnight at 4 °C with the primary antibodies. The following primary antibodies were utilized: anti-tyrosine hydroxylase (TH; mouse, 1:100; sc-25269, Santa Cruz Biotechnology, Dallas, TX, USA), anti-p-α-synSer129 (rabbit, 1:200; ab51253, Abcam, Cambridge, UK), and anti-MECR (rabbit, 1:200; ab254707, Abcam, Cambridge, UK). Following PBS washes, sections were incubated with fluorescently labeled secondary antibodies for 1 h in the dark. The secondary antibodies used were as follows: Alexa Fluor 488-conjugated donkey anti-mouse IgG (H + L) (1:400; Jackson ImmunoResearch, West Grove, PA, USA) and Cy3-conjugated donkey anti-rabbit IgG (H + L) (1:400; Jackson ImmunoResearch). After the final washes, the slices were prepared using an antifade mounting medium that included DAPI (Beyotime) and then covered with coverslips.

Visualization was performed using a confocal microscope (FV1200; Olympus, Tokyo, Japan). TH+ neurons were quantified following previously described methods [50]. Co-staining images of TH and MECR proteins were captured at 60× magnification with 8 z-stacks. For quantification of the average fluorescence intensity of TH+MECR+ cells, DA neurons were identified by setting a fixed threshold in the TH channel using Fiji (ImageJ 2.9.0), and the average fluorescence intensity of MECR in DA neurons was subsequently calculated.

### 4.11. Allosteric Modulator Screening, Docking, and MD

To screen for allosteric modulators of MECR, the MECR crystal structure (PDB ID: 7AYB) was utilized as the receptor protein. The CorrSite module of CavityPlus (http://pkumdl.cn:8000/cavityplus/, accessed on 8 October 2024) was employed to predict the allosteric binding pockets of MECR [51], selecting appropriate pockets based on Druggability and DrugScore.

The Protein Preparation Wizard module in Schrödinger (Schrödinger, New York, NY, USA) was used to prepare the protein, including element annotation corrections, addition of hydrogen atoms, bond order assignment, hydrogen bond optimization, and energy minimization using the OPLS4 force field. The processed protein was used for subsequent grid generation. Grid generation was executed through the Receptor Grid Generation panel of the Schrödinger Glide module (Release 2023-2), focusing on the allosteric site to ensure docking accuracy and binding site effectiveness.

Virtual screening was conducted using the small molecule library provided by ChemDiv database (https://www.chemdiv.com/complete-list/, 11 December 2024) with Schrödinger’s Virtual Screening Workflow (VSW) module. Initially, high-throughput virtual screening (HTVS) rapidly filtered compounds, with the top 10% by score advancing to standard precision (SP) docking. The top 10% from SP screening then proceeded to the extra precision (XP) docking stage to refine the selection of promising candidates. To enhance the accuracy of binding affinity predictions, Molecular Mechanics Generalized Born Surface Area (MM-GBSA) was applied to re-rank the XP docking results, calculating the binding free energy of each complex for re-evaluation and ranking. Following virtual screening and MM-GBSA re-ranking, the stability and conformational strain of each ligand were assessed by applying State Penalty and Ligand Strain Energy parameters. Compounds with high strain energy or excessive state penalties were excluded, ensuring that the final selections not only demonstrated high binding affinity but also exhibited stability and potential for drug development. After completing the threshold-based selection, further manual screening was conducted to rule out compounds that, although meeting numerical criteria, exhibited unfavorable interactions within the binding site. By analyzing hydrogen bonding, hydrophobic interactions, and other non-covalent interactions between ligands and the protein, we ensured that each selected compound maintained optimal spatial and chemical compatibility within the binding site.

MD simulations were then performed on the virtually screened compounds using Desmond software (Schrödinger Release 2023–2) to assess the stability of the compound–protein interactions. An orthorhombic simulation box was configured with boundaries at least 10 Å from the MECR–compound complexes. Physiological conditions were simulated by adding 150 mM NaCl, with counterions introduced to achieve charge neutrality in the solvated system. The entire system underwent energy minimization to 1 kcal mol^−1^ Å^−1^ using the OPLS4 force field. The minimized system was then subjected to a 100 ns MD simulation under normal temperature and pressure ensemble conditions at 300 K and atmospheric pressure, with default relaxation settings applied before the simulation began.

### 4.12. Statistical Analysis

Statistical analyses were conducted using R version 4.4.1 for bioinformatics analysis and GraphPad Prism 9 for cellular and animal experiments. Two-group comparisons were performed using either a parametric two-tailed unpaired Student’s *t*-test or a non-parametric Wilcoxon test. For multi-group comparisons, one-way analysis of variance (ANOVA) followed by Bonferroni’s post hoc test was applied. Statistical significance was set at *p* < 0.05.

## 5. Conclusions

This study employed transcriptomics and machine learning to identify MECR as a key lipid metabolism gene that is downregulated in PD. The downregulation of MECR was further validated in both cellular and animal models. Additionally, computational simulations identified potential allosteric modulators of MECR, providing a foundation for future studies and the development of new therapeutic approaches for PD.

## Figures and Tables

**Table 1:** Docking and binding analysis of six candidate compounds.

Compound ID (Internal)	Compound Name	State Penalty	Docking Score	Lig Strain Energy	MMGBSA dG Bind
M-01	3-[4-(3-chloro-4-methoxyphenyl)-3-oxopyrazin-2-yl]-1-(2-phenylethyl)urea	0	−4.86	3.02	−53.84
M-02	N-(carbamoylmethyl)-2-[7-(methoxymethyl)-3-oxo-5-phenyl-[1,2,4]triazolo[4,3-c]pyrimidin-2-yl]acetamide	0	−5.094	9.256	−53.02
M-03	3-(3-chloro-4-fluorophenyl)-1-[3-({7-methyl-4-oxopyrido[1,2-a]pyrimidin-2-yl}methoxy)phenyl]urea	0	−4.709	4.631	−52.22
M-04	3-(3-chlorophenyl)-1-[3-({7-methyl-4-oxopyrido[1,2-a]pyrimidin-2-yl}methoxy)phenyl]urea	0	−5.102	4.504	−52.02
M-05	3-[4-(3-chloro-4-methylphenyl)-3-oxopyrazin-2-yl]-1-(3-fluorophenyl)urea	0	−4.653	1.601	−51.64
M-06	me-thyl2-({5-[(carbamoylmethyl)sulfanyl]-2-sulfanylidene-1,3-dithiol-4-yl}sulfanyl)acetate	0	−4.832	2.868	−50.91

## Data Availability

Data are contained within the article and Supplementary Materials.

## Supplementary Materials

The following supporting information can be downloaded at: https://www.mdpi.com/article/10.3390/ijms26020550/s1.
