# Supplementary material for: Identification of Downregulated MECR Gene in Parkinson’s Disease Through Integrated Transcriptomic Analysis and Validation
Source: Int J Mol Sci. 2025 Jan 10;26(2):550. doi: 10.3390/ijms26020550 (PMC11765974; doi:10.3390/ijms26020550)
Supplement: Supplementary file 1 [file ijms-26-00550-s001.zip › Supplementary materials.pdf]

Supplementary Figures:

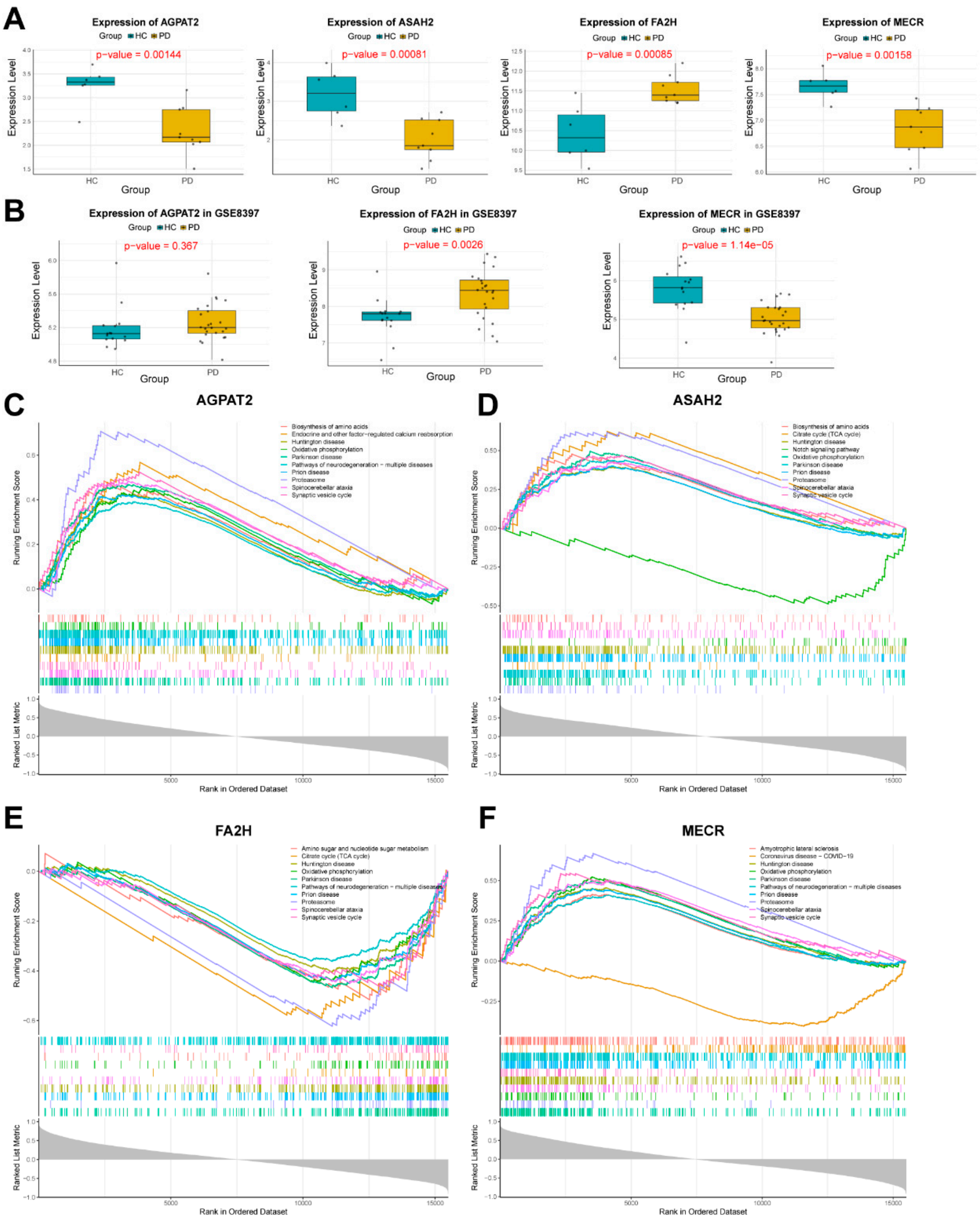

Figure S1. Gene expression and gene set enrichment analysis (GSEA) for AGPAT2, ASAH2, FA2H and MECR. (**A**) Gene expression of AGPAT2, ASAH2, FA2H and MECR in GSE42966. (**B**) Gene expression of AGPAT2, FA2H and MECR in GSE8397. (**C-F**) The top 10 pathways most correlated with genes AGPAT2 (**C**), ASAH2 (**D**), FA2H (**E**) and MECR (**F**) in GSEA based on KEGG database in GSE42966, ranked by normalized enrichment score (NES).

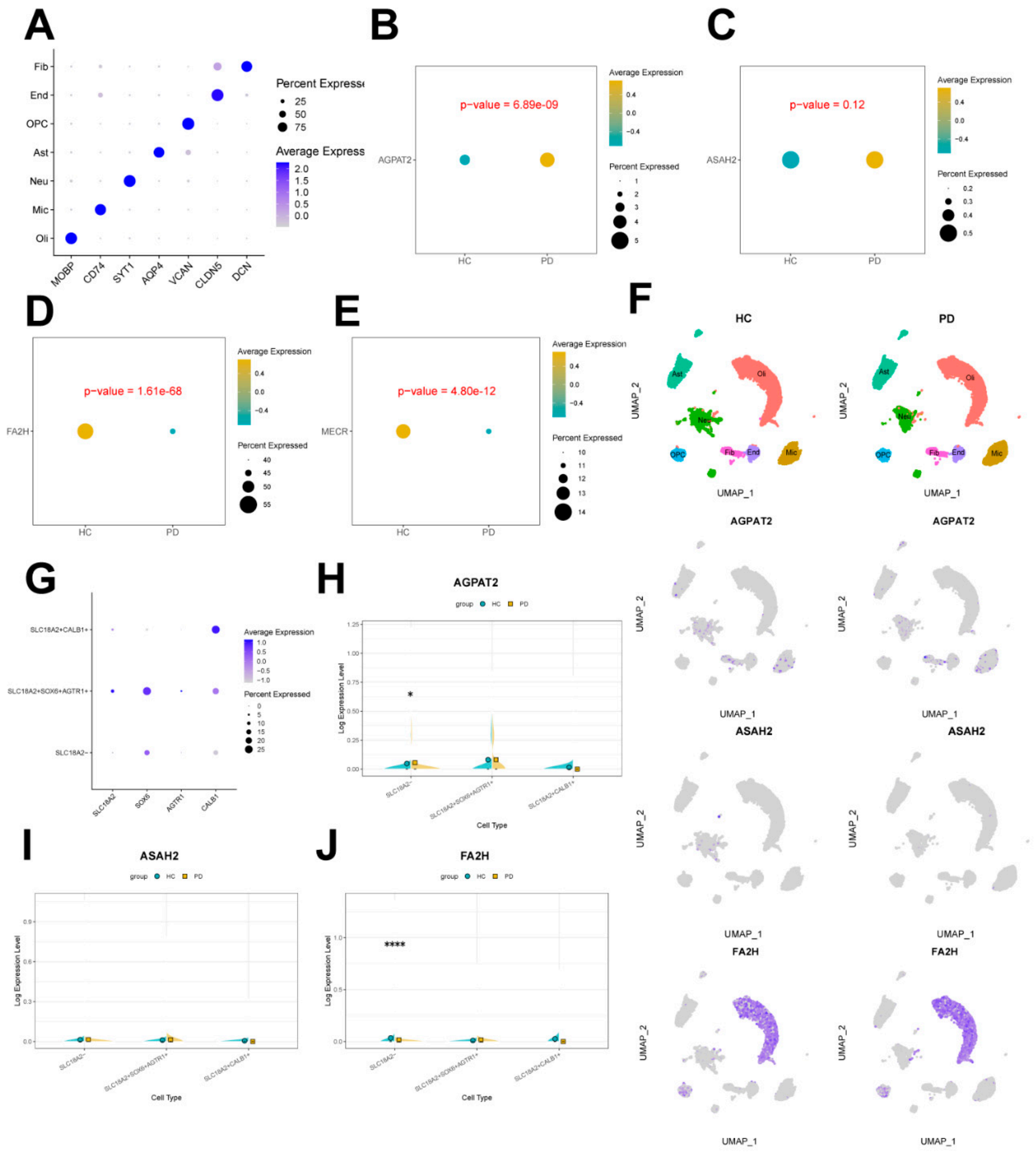

Figure S2. Candidate key genes expression in GSE157783 scRNA-seq dataset. (**A**) Dot plot of maker genes expression in seven cell types: oligodendrocytes (Oli), microglia (Mic), neurons (Neu), astrocytes (Ast), oligodendrocyte precursor cells (OPC), endothelial cells (End), and fibroblasts (Fib). (**B-E**) Dot plots showing differential expression of AGPAT2 (**B**), ASAH2 (**C**), FA2H(**D**) and MECR(**E**) between healthy controls (HC) and Parkinson's disease (PD) groups in GSE157783. (**F**) Feature plots showing AGPAT2, ASAH2 and FA2H expression across different cell types between HC and PD groups. (**G**) Dot plot of maker genes expression in three neuron subtypes: CALB1+ dopaminergic (DA) neurons (SLC18A2+CALB1+), SOX6+AGTR1+ DA neurons (SLC18A2+SOX6+AGTR1+) and non-DA neurons (SLC18A2-). (**H-J**) Violin plots demonstrating differential expression of AGPAT2 (H), ASAH2 (I) and FA2H(J) between PD and HC groups across three neuron subtypes. Significance was tested using Wilcoxon Rank Sum test. *p < 0.05, ****p < 0.001.

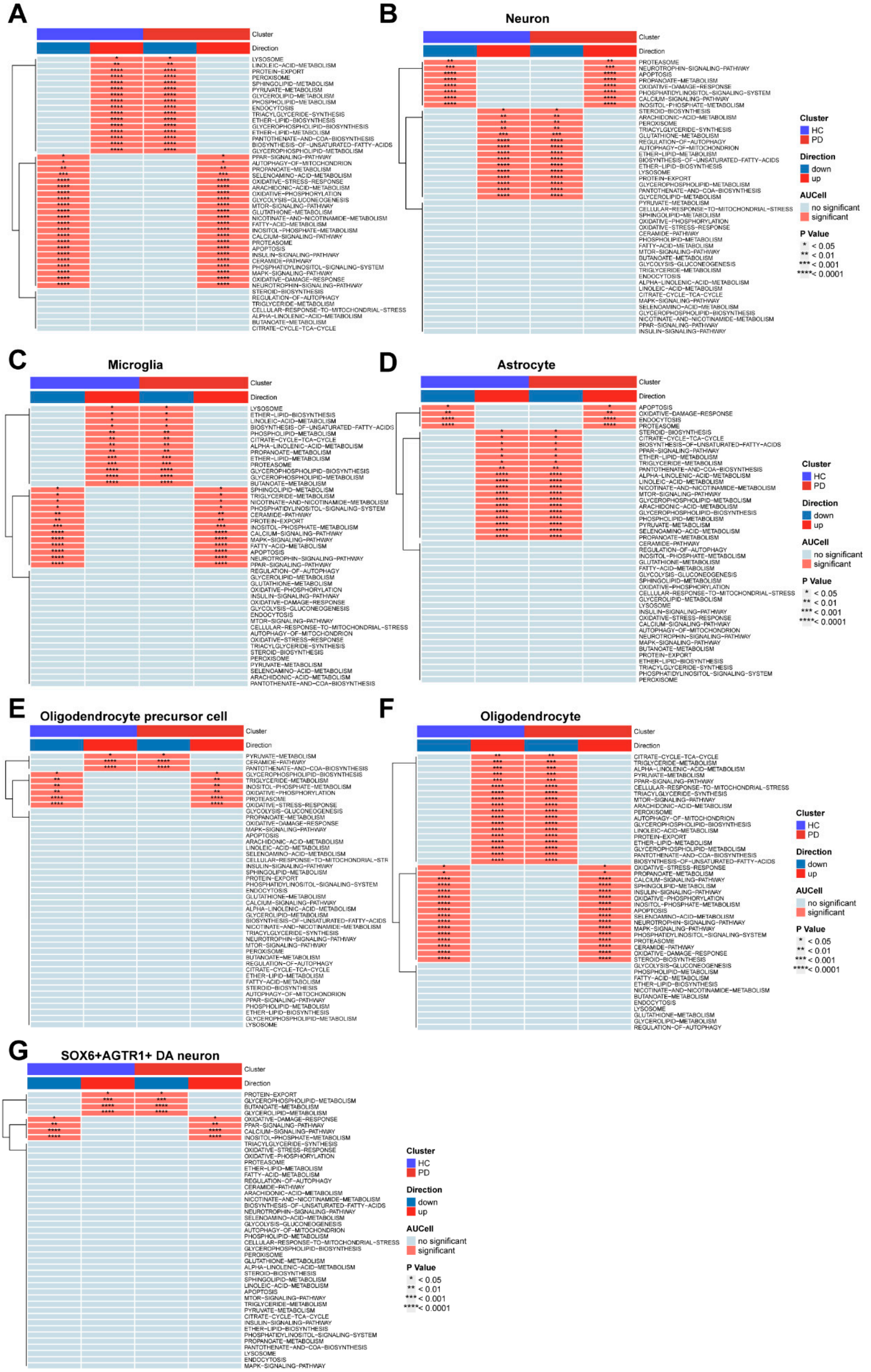

Figure S3. Lipid metabolic reprogramming across cell types in PD SN. (**A**) The heatmap shows overall

upregulation and downregulation trends in lipid metabolism pathways between PD and healthy control (HC) groups. (**B-G**) Heatmaps displaying the specific upregulation or downregulation of lipid synthesis and metabolism pathways across different cell types, including neurons (**B**), microglia (**C**), astrocytes (**D**), oligodendrocyte progenitor cells (OPCs) (**E**), oligodendrocytes (**F**), and SOX6+AGTR1+ dopaminergic (DA) neurons (**G**).

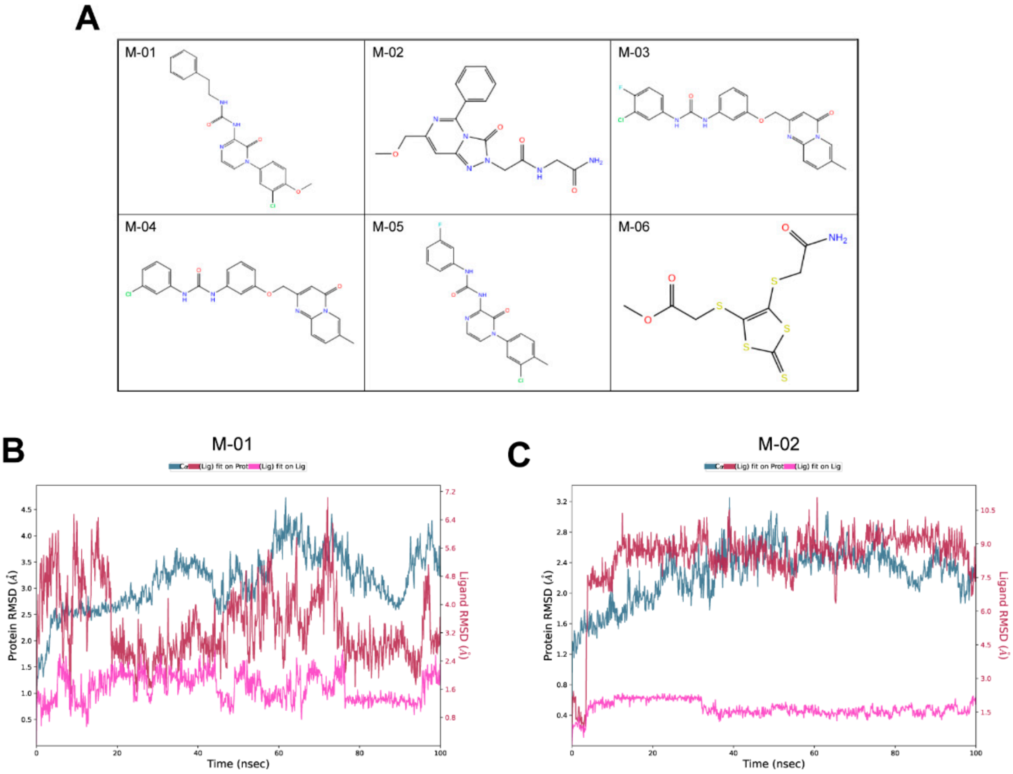

Figure S4. (**A**) Chemical structures of the six candidate drugs. (**B**) Protein-ligand RMSD plot of compound M-01. (**C**) Protein-ligand RMSD plot of compound M-02.

Supplementary Videos:

Video S1: Molecular dynamics simulations of MECR protein binding to M-03.

Video S2: Molecular dynamics simulations of MECR protein binding to M-01.

Video S3: Molecular dynamics simulations of MECR protein binding to M-02.
